# Rapid detection of porcine circovirus type 2 using a TaqMan-based real-time PCR

**DOI:** 10.1186/1743-422X-7-374

**Published:** 2010-12-31

**Authors:** Kai Zhao, Fangting Han, Yong Zou, Lianlong Zhu, Chunhua Li, Yan Xu, Chunling Zhang, Furong Tan, Jinbin Wang, Shiru Tao, Xizhong He, Zongqing Zhou, Xueming Tang

**Affiliations:** 1 Biotechnology Research Institute, Shanghai Academy of Agricultural Sciences, 2901 Beidi Road, Shanghai, 201106, People's Republic of China; 2 Institute of Animal Science and Veterinary Medicine, Shanghai Academy of Agricultural Sciences, 2901 Beidi Road, Shanghai, 201106, People's Republic of China; 3 Key Laboratory of Agricultural Genetics and Breeding, Shanghai Academy of Agricultural Sciences, 2901 Beidi Road, Shanghai, 201106, People's Republic of China; 4 College of Life and Environment Sciences, Shanghai Normal University,100 Guilin Road, Shanghai 200234, People's Republic of China

## Abstract

Porcine circovirus type 2 (PCV2) and the associated disease postweaning multisystemic wasting syndrome (PMWS) have caused heavy losses in global agriculture in recent decades. Rapid detection of PCV2 is very important for the effective prophylaxis and treatment of PMWS. To establish a sensitive, specific assay for the detection and quantitation of PCV2, we designed and synthesized specific primers and a probe in the open reading frame 2. The assay had a wide dynamic range with excellent linearity and reliable reproducibility, and detected between 10^2 ^and 10^10 ^copies of the genomic DNA per reaction. The coefficient of variation for Ct values varied from 0.59% to 1.05% in the same assay and from 1.9% to 4.2% in 10 different assays. The assay did not cross-react with porcine circovirus type 1, porcine reproductive and respiratory, porcine epidemic diarrhea, transmissible gastroenteritis of pigs and rotavirus. The limits of detection and quantitation were 10 and 100 copies, respectively. Using the established real-time PCR system, 39 of the 40 samples we tested were detected as positive.

## Introduction

Porcine circovirus type 2 (PCV2) is widespread in the commercial swine population [1-5], and is accepted as the causative agent of a number of diseases in these animals, particularly postweaning multisystemic wasting syndrome (PMWS) [6]. To date, PCV2 infection is common in some regions of China [7], and is considered as a major problem in pig production. There is therefore an urgent need for specific and effective methods to detect the virus.

By comparison with conventional PCR and ELISA, real-time PCR offers an effective way to detect target fragments specifically, rapidly and quantitatively. False-positive results and pollution can be prevented effectively at the same time. Therefore, real-time PCR has been developed quickly and has become the main method for pathogen detection [8].

In this study, we designed and synthesized specific primers and a TaqMan probe for PCV2. We have established an assay that is specific and sensitive for detection and quantitation of PCV2.

## Materials and methods

### Design of primers and TaqMan probe

The primer and TaqMan probe design were based on nucleotide sequences of open reading frame 2 (ORF2) retrieved from GenBank (EU921257.1), using the PCV2 strain from China (BJ0804) as a master sequence. The primers and probe (Table 1) were designed using Primer Premier 5.0, Oligo Primer Analysis software and DNAman 4.0. The length of the amplified product was 149 bp.

**Table 1:** Sequences of primers and probe of PCV2

Primes and probe	Sequence
Primer-1	5'-CGGATATTGTAKTCCTGGTCGTA-3'
Primer-2	5'-CCTGTCCTAGATTCCCCTATTGATT-3'
Probe	FAM-5'-CTAGGCCTACGTGGTCTACATTTC-3'-TAMRA

### Preparation of standard plasmid DNA

The standard plasmid was constructed by inserting a PCR fragment into a pGEM-T Easy vector according to the manufacturer's instructions (Promega, Madison, WI, USA). The plasmid was propagated in *Escherichia coli *JM109 cells and was purified and subsequently quantified using an ND-1000 spectrophotometer (NanoDrop, Wilmington, DE, USA). Ten-fold dilutions were made to obtain 10^10^-10^0 ^per μL plasmid sample (containing 100 ng/μL yeast tRNA) for the real-time PCR. The dilutions were stored at -20°C, while the plasmids were stored at -70°C.

### Conventional PCR reaction

PCR amplifications were performed in 25-μL reaction volumes containing 1×PCR buffer, 200 μM dATP, dTTP, dCTP and dGTP, 1.25 U DNA polymerase, 2 mM MgCl_2 _(TaKaRa, Dalian, China), 200 nM of each primer, and different quantities of the plasmid DNA templates. Amplifications were programmed as follows: one step of 94°C for 5 min, 30 cycles of 94°C for 30 s, 60°C for 20 s and 72°C for 20 s, and one step of 72°C for 7 min. Amplicons of 149 bp were separated through 2% agarose gel containing 5% Goldview (SBS Genetech, Shanghai, China). Negative and positive reference samples were applied in each reaction.

### TaqMan real-time PCR

Real-time PCR was carried out on an ABI 7500 thermocycler (Applied Biosystems, CA, USA) with a final volume of 25 μL. The real-time PCR reactions contained the following ingredients: 1×PCR buffer, 400 nM primers, 200 nM TaqMan probes, 400 μM each of dATP, dTTP, dGTP and dCTP, 1.25 U Taq DNA polymerase, and 4.5 mM MgCl_2_. Real-time PCR reactions were run as follows: 95°C for 10 min and 45 cycles of 95°C for 15 s and 60°C for 40 s. For a standard curve, serial dilutions of 10^10 ^to 10^0 ^copies of the plasmid were used. Each assay was performed in duplicate and each run included two negative controls.

### Limits of detection and quantitation of the assay

To establish the limit of quantitation (LOQ) of the assay, samples containing 10^7^, 10^5^, 10^3 ^and 10^2 ^copies per sample were run in triplicate, and samples containing 90, 80, 70, 60, 50, 40, 30 and 20 copies were also included. Samples containing 10 copies and one copy per sample were also run to estimate the limit of detection (LOD) of the assay.

### Reproducibility and specificity of the assay

The standard PCV2 plasmid with 10^7^, 10^5 ^and 10^3 ^copies was used to evaluate the coefficients of variation (CVs) of the real-time PCR. Intra- and inter-assay CVs for Ct values were both included. To test the specificity of the assay, plasmid samples containing 10^8^, 10^7^, 10^6^, 10^5 ^and 10^4 ^copies together with cDNA of porcine reproductive and respiratory, porcine epidemic diarrhea, transmissible gastroenteritis of pigs and rotavirus and DNA of porcine circovirus type 1 were run under optimal conditions of the assay. Negative controls were also contained in the run.

### Detection of clinical samples

Three PCV2-positive samples and 37 serum and tissue unknown samples were tested using conventional PCR and real-time PCR under optimal conditions. Products from conventional PCR were examined in 2% agarose gel.

## Results

### Real-time PCR for PCV2 DNA

**Figure 1:**
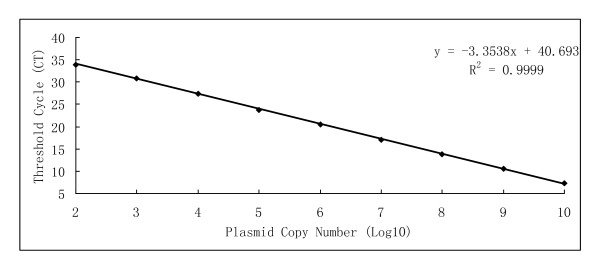
**Standard curve between Ct value and log_10 _copy number of PCV2 plasmid DNA**.

Ten-fold serial plasmid dilutions were used to construct the standard curve by plotting the logarithm of the plasmid copy number against the measured Ct values (Figure 1). The standard curve generated had a wide dynamic range of 10^2^-10^10 ^copies/μL with a linear correlation (*R*^2^) of 0.9999 between the Ct value and the logarithm of the plasmid copy number.

### LOD and LOQ of the assay

**Figure 2:**
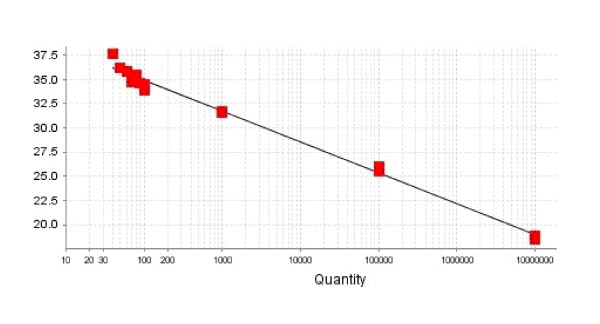
**Determination of the limit of quantitation**.

**Figure 3:**
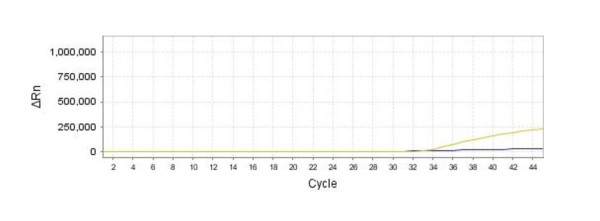
**Determination of the limit of detection**. The yellow and blue curves reveal the fluorescence values observed in samples containing 10 and 1 copies of PCV2 plasmid respectively.

For reliable quantitation of the results under ideal conditions, approximately 100 initial template copies were required, thereby specifying the LOQ of this assay. When the number of template copies fell below 100, the Ct values lay outside of the linear range (Figure 2). The target sequence could be detected in all amplification reactions down to 10 copies, but not when only one copy was present (Figure 3). These results indicate that the LOD value was ~10 copies.

### Reproducibility and specificity of the assay

The CVs for the Ct values ranged from 0.59% to 1.05% in the same assay and from 1.9% to 4.2% in 10 different assays (Table 2). No increase in fluorescence was observed in the negative control and PCV1, PRRS, PED, TGE and RV samples.

**Table 2:** Intra- and inter-assay reproducibility of the real-time PCR

Concentration of plasmid Standard (copy/μl)	Intra-assay			Inter-assay		
	
	Mean Ct	SD	CV (%)	Mean Ct	SD	CV (%)
107	17.88	0.1069	0.68	17.69	0.7498	4.2
105	24.49	0.2589	1.05	24.03	0.5597	2.3
103	30.98	0.2092	0.59	30.22	0.5632	1.9

SD, standard deviation; CV, Coefficient of variation

### Detection of clinical samples

Table 3 and 4 showed that the PCV2-positive rates in the unknown samples of conventional PCR detection and real-time PCR detection were 78.3% and 97.3%, respectively. The real-time PCR approach increased the detection of PCV2 samples by 18% over that achieved by conventional PCR.

**Table 3:** Comparison between conventional PCR and real-time PCR for PCV2 positive samples

Conventional PCR	Real time PCR		Subtotal
		
	+	-	
+	3	0	3
-	0	0	0

Total			3

+, positive; -, negative

**Table 4:** Comparison between conventional PCR and real-time PCR for unknown samples

Conventional PCR	Real time PCR		Subtotal
		
	+	-	
+	29	0	29
-	7	1	8

Total			37

+, positive; -, negative.

The viral loads were mostly between 10 and 1000 copies/μL sample with a few samples containing up to 10^8 ^copies/μL. Three hundred and sixty and 1560 copies of PCV2 were detected per microliter in the PPV and PRV DNA extracted from serum samples. It appeared that the pigs from which the PPV and PRV DNA samples were obtained were co-infected with PCV2.

## Discussion

Serological surveys have shown that up to 100% of investigated farms and up to 100% of individual pigs sampled in parts of Europe, the United States and Canada are seropositive for PCV2 [9-11]. Using ELISA on samples collected in seven provinces and municipalities in China, the seropositive rate was found to be up to 42.9% [12].

PCV2-induced diseases on farms are reported to increase pig mortality from 2-3% to 14-30% [13]. Therefore, rapid and sensitive detection and quantitation assays for PCV2 are urgently needed both by the pig industry and research community. In comparison with conventional PCR, TaqMan real-time PCR is more sensitive and less easily contaminated. The main difficulty of using conventional PCR is that contamination occurs when products are examined in gels, which leads to false-positive results in later experiments. For this reason, real-time PCR is widely used, and in addition, it has heightened sensitivity and requires less time than conventional PCR.

The major conserved region for PCV2 located in ORF2 is likely to be the ideal reference fragment to detect PCV2, because this region displays the highest diversity between PCV1 and PCV2 and there are more sequenced isolates available from PCV2 than there are from PCV1 [14]. Hybridization probes that combine only with the target products have primarily been used in previous studies to detect PCV2, and the results of these studies have shown high sensitivity and specificity. Several other methods are available to detect and quantify PCV2. Brunborg et al. [14] have used a TaqMan probe to detect an 84-bp fragment in ORF2 and to quantify the viral load in different tissues and serum samples. In a report by Chung et al. [15], PCV2 was quantified in naturally infected and challenged pigs using a TaqMan real-time PCR that detected a fragment of 269 bp. Yang et al. [16] have used SYBR Green I based on nucleotide sequences of ORF2 for the detection of PCV2.

In this study we designed different primers, a different probe and a different real-time PCR system, which amplified a 149-bp fragment to detect PCV2. The real-time PCR approach increased the detection of PCV2 samples by 18% over that achieved by conventional PCR. Tests on the reproducibility of the method suggest that the established real-time PCR system appears to be reliable and stable. A series of experiments were carried out to assess the reproducibility, sensitivity, and specificity of the assay. Using several other swine viruses as template, no cross-reaction signals were detected, which demonstrated the specificity of the assay. The established real-time PCR system that we developed might not only provide an effective way to detect PCV2 rapidly and sensitively, but might also be applied to assess the effectiveness of vaccines developed to combat PCV2. The real-time PCR detection system complements and extends previous methods for detection and quantitation of PCV2. The specific detection method can also provide an alternative approach for detection of PCV2.

## Abbreviations

bp: base pair; cDNA: complementary DNA; LOD: limit of detection; LOQ: limit of quantitation; ORF2: open reading frame 2; PCV1: Porcine circovirus type 1; PCV2: Porcine circovirus type 2; PED: Porcine epidemic diarrhea; PMWS: Postweaning multisystemic wasting syndrome; PPV: Porcine parvovirus; PRRS: Porcine reproductive and respiratory; PRV: Pseudorabies virus; RV: Rotavirus; TGE: Transmissible gastroenteritis of pigs;.

## Competing interests

The authors declare that they have no competing interests.

## Authors' contributions

KZ, FH and XT participated in the design and carried out the majority of the experiments in the study and drafted the manuscript. YZ, LZ, CL, YX, CZ, FT, JW, ST, XH, ZZ and XT helped to carry out the experiments and draft the manuscript. All authors read and approved the final manuscript.
